# Anti-inflammatory benefits of semaglutide: State of the art

**DOI:** 10.1016/j.jcte.2024.100340

**Published:** 2024-03-28

**Authors:** Habib Yaribeygi, Mina Maleki, Tannaz Jamialahmadi, Amirhossein Sahebkar

**Affiliations:** a Research Center of Physiology, Semnan University of Medical Sciences, Semnan, Iran; b Urology and Nephrology Research Center, Shahid Beheshti University of Medical Sciences, Tehran, Iran; c Medical Toxicology Research Center, Mashhad University of Medical Sciences, Mashhad, Iran; d Pharmaceutical Research Center, Pharmaceutical Technology Institute, Mashhad University of Medical Sciences, Mashhad, Iran; e Applied Biomedical Research Center, Mashhad University of Medical Sciences, Mashhad, Iran; f Biotechnology Research Center, Pharmaceutical Technology Institute, Mashhad University of Medical Sciences, Mashhad, Iran

**Keywords:** GLP-1, Semaglutide, Diabetes mellitus, Insulin resistance, Inflammation

## Abstract

Individuals with diabetes often have chronic inflammation and high levels of inflammatory cytokines, leading to insulin resistance and complications. Anti-inflammatory agents are proposed to prevent these issues, including using antidiabetic medications with anti-inflammatory properties like semaglutide, a GLP-1 analogue. Semaglutide not only lowers glucose but also shows potential anti-inflammatory effects. Studies suggest it can modulate inflammatory responses and benefit those with diabetes. However, the exact mechanisms of its anti-inflammatory effects are not fully understood. This review aims to discuss the latest findings on semaglutide's anti-inflammatory effects and the potential pathways involved.

## Introduction

The global prevalence of diabetes mellitus (DM) is rapidly increasing [1]. This chronic metabolic disorder is characterized by elevated blood glucose levels and is associated with various metabolic complications and harmful pathways affecting lipids and carbohydrates [2]. Within the context of diabetes, several detrimental pathways, such as oxidative stress, inflammation, necrosis, and fibrosis, are activated and exacerbated [2]. Consequently, these pathways can cause damage to cells and tissues, leading to disability or even death [2], [3]. As a result, DM is now recognized as a significant risk factor for severe complications, driving the development of herbal and synthetic antidiabetic treatments aimed at managing the disease and alleviating its complications [3], [4], [5], [6]. Despite these efforts, effectively controlling injurious pathways, such as inflammation, in the diabetic environment remains a considerable challenge [7], [8].

Semaglutide is an approved medication belonging to the class of incretin-based therapies for individuals with type 2 (T2)DM [9]. It has demonstrated potent antidiabetic effects and effectively lowers blood glucose levels through multiple cellular pathways [9], [10]. Recent evidence suggests that semaglutide, like other glucagon-like peptide-1 (GLP-1) receptor agonists [11], [12], [13], [14], [15], [16], may offer additional benefits beyond glycemic control and can suppress certain harmful pathways [17], [18], [19]. However, the precise impact of semaglutide on inflammatory responses, a major pathophysiologic pathway implicated in diabetic complications [8], [20], is not yet fully understood. In this mechanistic review, our objective is to explore the potential benefits of semaglutide in mitigating inflammatory responses.

## Classifications of diabetes mellitus

DM is commonly classified into four main types [21]. T1DM, characterized by a deficiency of circulating insulin due to beta cell dysfunction or failure [21]. T2DM, the most prevalent form of DM, primarily associated with insulin resistance in peripheral tissues [21]. Gestational diabetes occurs in pregnant women and is believed to be caused by hormonal changes [16]. Additionally, less frequently occurring forms of DM include latent autoimmune diabetes in adults (LADA), maturity-onset diabetes of the young (MODY), and secondary diabetes resulting from conditions like pancreatitis or certain medications, such as corticosteroids. These specific forms collectively form the fourth category of DM [21], [22], [23].

## Inflammation, roles in diabetic complications

Chronic hyperglycemia, characterized by high blood sugar levels, is strongly associated with increased incidence of inflammatory reactions [24], [25], [26]. These inflammatory reactions play a significant role in the development of diabetes mellitus and its associated complications [27], [28]. Elevated glucose levels can activate immune cells and trigger the release of inflammatory cytokines, making chronic hyperglycemia a key driver of inflammation in diabetes [29]. Inflammation is involved in the pathophysiology of insulin resistance and diabetes, disrupting insulin signal transduction [10]. Additionally, mounting experimental and clinical evidence confirms that inflammation is implicated in the pathophysiology of diabetes-induced vascular disorders, including diabetic retinopathy, diabetic nephropathy, diabetic neuropathy, and cardiovascular disorders [8], [27], [28], [30]. It also contributes to other diabetic complications, such as fatty liver [31]. Moreover, patients with diabetic complications typically exhibit elevated levels of inflammatory cytokines in their plasma [28], [32].

Numerous inflammatory mediators, including tumor necrosis factor-alpha (TNF-α), interleukins (IL-1β, IL-6, IL-18), matrix metalloproteinases (MMPs), chemokine ligand 2 (CCL-2), monocyte chemoattractant protein-1 (MCP-1), nuclear factor kappa B (Nf-κB), transforming growth factor-beta (TGF-β), E-selectin, various adhesion molecules (ICAM-1, VCAM-1), toll-like receptors (TLRs), adiponectin, endothelial cell-selective adhesion molecule (ESAM), and interferon-gamma (INF-γ), are strongly implicated in diverse forms of diabetic complications [33], [34], [35]. Furthermore, these potent biological elements are highly expressed and secreted in the diabetic context, supporting the “inflammation theory” that emphasizes the pivotal roles of inflammatory responses in the pathophysiology of diabetes mellitus and its associated complications [28], [36]. Therefore, understanding and addressing the inflammatory component of diabetes is crucial for the development of effective therapeutic strategies aimed at preventing or mitigating complications associated with this disease.

## GLP-1 receptor agonists and semaglutide

**Fig. 1:**
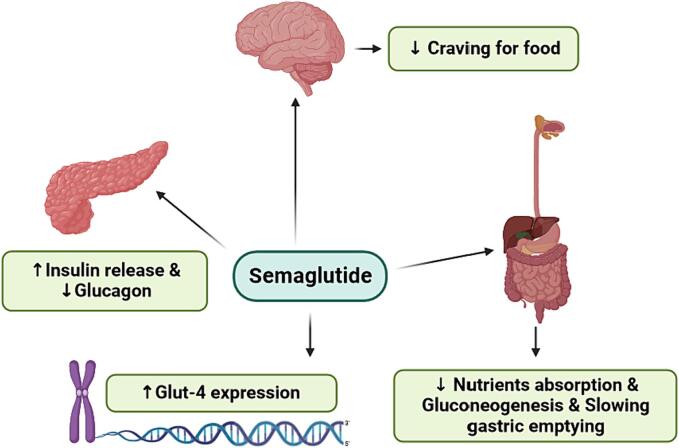
Semaglutide modulates post-prandial glucose levels thru several pathways.

Incretin-based medications are a class of drugs commonly used in the management of T2DM (Table 1) [37]. These medications target the incretin system, which plays a crucial role in regulating blood sugar levels [37], [38]. Incretins are hormones released by the enteroendocrine L-cells of the gastrointestinal (GI) tract in response to food intake [38]. They stimulate the release of insulin from the pancreas and reduce the production of glucagon, helping to normalize postprandial glucose levels [38], [39]. Incretin-based medications mimic the actions of natural incretins, such as GLP-1 and gastric inhibitory hormone (GIP). They increase insulin secretion from the pancreas, decrease glucose production by the liver, slow down stomach emptying, and suppress appetite [10]. There is also evidence suggesting that GLP-1 up-regulates insulin expression [40] (Fig. 1).

**Table 1:** Pharmacological properties of the main approved forms of GLP-1 receptor agonists.

	**Name**	**Half life**	**Administration**	**Ref.**
**GLP-1 RA**	Exenatide	2.4 h	Twice daily subcutaneously	[45], [46], [47]
	Exenatide (extended-release)	–	Once weekly subcutaneously	[45], [46], [48], [49]
	Liraglutide	13 h	Once daily subcutaneously	[45], [46], [50]
	Albiglutide	4–7 days	Once weekly subcutaneously	[45], [46], [51]
	Dulaglutide	5 days	Once weekly subcutaneously	[45], [46], [52]
	Lixisenatide	3 h	Once daily subcutaneously	[45], [46], [53]
	Semaglutide	168 h	One weekly subcutaneously or once daily orally	[45], [46], [54]

GLP-1, a 30-amino acid peptide, is produced from the pre-proglucagon within enteroendocrine L-cells located in the GI tract [41]. GLP-1 receptor agonists (GLP-1RAs) are a category of antidiabetic medications designed to induce hypoglycemic effects by replicating the actions of incretin hormones, particularly through the activation of GLP-1 receptors [41], [42]. These medications act on the GLP-1 receptor, a type of G-protein coupled receptor primarily found on the surfaces of pancreatic beta cells [42]. Activation of the GLP-1 receptor leads to the generation of cyclic adenosine monophosphate (cAMP), subsequent cellular depolarization, and insulin secretion from pancreatic beta cells in response to feeding [42], [43], [44].

Semaglutide is a specific type of incretin-based medication known as a GLP-1 analogue. It binds to its specific receptors on pancreatic beta cells, enhancing insulin secretion in response to postprandial hyperglycemia (Fig. 1) [42], [55]. It also inhibits glucagon release, which helps reduce excessive glucose production by the liver [55]. Additionally, semaglutide can promote a feeling of fullness, reduce food cravings, and lead to a reduction in food intake and, consequently, weight loss in some individuals [55], [56]. There is also evidence suggesting that semaglutide induces the expression of Glut-4 [57]. Semaglutide is often administered as an injectable medication, but it is also available in an oral form, making it the only oral GLP-1 analogue currently available [9], [58]. Three forms of semaglutide, namely Ozempic, Rybelsus, and Wegovy, have been approved by the FDA [59], [60]. Like all synthetic drugs, semaglutide may induce some adverse effects, such as nausea and diarrhea [61].

## Semaglutide and inflammation

While semaglutide is primarily known for its metabolic benefits, such as regulating glucose levels, promoting weight loss, and normalizing lipid profiles, there is emerging evidence suggesting that it may also have additional anti-inflammatory effects [20], [62]. In fact, there is strong evidence indicating that semaglutide can modulate or reduce inflammatory processes [63], [64], [65]. Considering that inflammation is a key factor in many diabetic complications, these anti-inflammatory effects of semaglutide could provide additional benefits, particularly in the cardiovascular system [66]. Therefore, semaglutide may have protective roles in addition to its metabolic benefits, potentially benefiting the cardiovascular system [66], [67], liver tissue [68], and kidneys [69].

Although there is still limited evidence exploring the specific mechanisms involved, current knowledge suggests two major pathways by which semaglutide exerts its anti-inflammatory effects: reducing inflammatory cytokine levels and modifying immune system activity. It is important to note that these pathways may overlap and be interconnected in many cases. In the following sections, we will present the latest findings regarding the anti-inflammatory roles of semaglutide and discuss the possible mechanisms involved, drawing from both clinical trials and experimental studies.See (Table 2).

**Table 2:** Pharmacokinetic properties of oral and injective Semaglutide.

	Oral Semaglutide	Injective Semaglutide
Absorption		
Bioavailability	0.5–1 %	89 %
Steady state plasma level	14.6 nmol/L (14 mg once daily)	123 ng/ml (1 mg weekly once)
Time to achieve steady state level	4–5 weeks	4–5 weeks
Time to achieve maximum level	1 h	1–3 days
Distribution		
Protein binding	> 99 %	> 99 %
Metabolic pathway	Proteolytic degradation followed by fatty acid oxidation	
Volume of distribution	8 Liters	12.5 Liters
Elimination profile		
Elimination t1/2	7 days	7 days
Rate of clearance	0.04 L/hour	0.05 L/hour

### Reducing the inflammatory cytokines

Semaglutide, a GLP-1 receptor agonist, has been shown to have anti-inflammatory effects by suppressing the release of pro-inflammatory cytokines, such as IL-6 and TNF-α [63], [70]. In an animal model of seizures using pentylenetetrazole, semaglutide demonstrated neuroprotective effects and improved cognitive function by inhibiting the release of inflammatory cytokines mediated by the NLRP3 inflammasome, a complex involved in regulating the innate immune system and inflammatory responses. This effect was observed in mice [70].

In another animal seizure model, semaglutide reduced inflammation signaling pathways, including p38 MAPK, c-Jun-Nf-κB p65, in brain tissues of rats [71]. These neuroprotective effects were also demonstrated in another experiment [72]. In a study using male Swiss albino mice, semaglutide reduced levels of TNF-α, IL-6, and IL-1β in brain tissues during endotoxemia and polymicrobial sepsis, leading to improved cognitive abilities [72]. Additionally, semaglutide reduced lung injury in a rat model of lipopolysaccharide (LPS)-induced acute lung injury by suppressing TNF-α, IL-6, and Nf-κB activities [73].

Clinical evidence also supports the anti-inflammatory effects of semaglutide. In a study involving 40 men with T2DM, treatment with 1 mg of semaglutide per week for 6 months reduced circulating levels of inflammatory cytokines TNF-α and IL-6 [63]. This effect may contribute to a reduction in systemic inflammation and potentially lower the risk of cardiovascular disorders [63]. A more recent clinical study reported anti-inflammatory effects of semaglutide (1 mg/week) in patients with T2DM, although these effects were not significant after 3 months [64]. Furthermore, a *meta*-analysis examining the effects of semaglutide on the inflammatory cytokine high-sensitive C-reactive protein (hsCRP) found a significant reduction in its levels in patients with T2DM [74].

In a recent clinical study, semaglutide improved renal function in patients with T2DM by reducing inflammatory responses [65]. It has also been suggested that semaglutide may have greater anti-inflammatory potential in suppressing the inflammatory storms induced by COVID-19 compared to other GLP-1 mimetics [75], [76]. Overall, the available evidence suggests that semaglutide has the ability to attenuate or block the release of inflammatory cytokines in various tissues [77] (Table 3, Table 4).

**Table 3:** Experimental studies suggesting anti-inflammatory properties of semaglutide (TNF-α = tumor necrosis factor alpha, IL-6 = interleukin-, Nf-κB = nuclear factor kappa b, NLRP3 = NLR family pyrin domain containing 3 inflammasome, MAPK = mitogen activated protein kinase, c-Jun = transcription factor Jun).

**Effects**	**Model**	**Treatment**	**Ref.**
Reduced the TNF-α, IL-6 and Nf-κB signalings	LPS-induced lung injury in rats	Semaglutide	[73]
Blocked the NLRP3 activity	PTT-induced seizure in C57/BL6J mouse	Semaglutide	[70]
Reduced the TNF-α, IL-6, and IL-1β levels in brain tissues	Endo-toxemia in male Swiss albino mice	Semaglutide	[72]
Reduced p38 MAPK, c-Jun- NF-κB p65 inflammation signaling pathway in brain tissues	Animal model of seizure	Semaglutide	[71]
Reduced intramuscular fat and improved muscle function by lowering the, TNF-α, IL-6, IL-1β levels	Male C57BL/6 mice	Semaglutide	[78]
Declined TNF-α, and IL-6 serum and heart tissues	Obese mouse	Semaglutide	[62]
Decreased vascular inflammation and micro-calcifications	Obese rabbit	Semaglutide	[79]
Attenuated inflammatory markers and improved cardiac function	Obese mice	Semaglutide	[80]

**Table 4:** Clinical or human evidences explored anti-inflammatory effects of semaglutide (CKD = chronic kidney disease, hsCRP = high-sensitive C-reactive protein).

**Treatment**	**Patients/samples**	**Dose/duration**	**Effects**	**Ref.**
Semaglutide	40 men with DM	1 mg/week/6 months/injection	Reduced the inflammatory cytokines of TNF-α and IL-6	[63]
Semaglutide	20 patients with T2DM	1 mg/week/3 months/injection	Minor changes in some inflammatory cytokines (not meaningful) e.g. CRP and IL-6	[64]
Semaglutide	Patients with T2DM	–	Semaglutide is associated to reduced levels of hsCRP vs baseline in patients with T2DM	[74]
Semaglutide	Obese patients with T2DM	0.25 mg/week for 4 weeks, increased to 0.50 mg/week for 16 weeks, and then to 1 mg/week for 10 months	Semaglutide improved psoriasis and epicardial fat volume and inflammation	[89]
Semaglutide	Patients with T2DM and CKD	3 mg/day/9months/orally	Semaglutide improved renal function probably by lowering inflammation	[65]
Semaglutide	Epicardial fat biopsies of patients undergoing open-heart surgery	–	Semaglutide reduced the neutrophils adhesion into endothelial cells and enhances the angiogenesis process	[67]
Semaglutide	Epicardial fat biopsies of patients undergoing cardiac surgery	–	Semaglutide induced anti-thrombotic and anti-atherosclerotic effects by suppressing neutrophils’ activity	[87]

### Modulation of immune system response

Semaglutide has the ability to modulate immune system activity through various pathways [62], [66], [81]. GLP-1 receptors are found on different immune cells, such as neutrophils and eosinophils [82], [83], [84], [85], and their activation has modulatory effects on immune responses and inflammatory processes [83], [84]. Evidence suggests that semaglutide can activate these receptors and modulate immune system activity [83], [86]. McLean et al. demonstrated that semaglutide activates GLP-1 receptors on endothelial and hematopoietic cells in mice [86]. They observed a subsequent reduction in inflammatory cytokines such as TNF-α, Abcg1, TGF-β1, Cd3g, and CCL-2 in hepatocytes [86]. Emerging evidence has also suggested similar benefits in epicardial fat [67].

A recent study reported that semaglutide decreases inflammatory processes in epicardial fat of patients undergoing open-heart surgery [67]. This study demonstrated that semaglutide reduces the activity of neutrophils and their adhesion to endothelial cells in human epicardial fat, which expresses GLP-1 receptors [67]. Another recent study provided further evidence suggesting that semaglutide suppresses neutrophil activation in epicardial fat collected from patients undergoing cardiac surgery [87]. Since the neutrophil-to-lymphocyte ratio is associated with cardiovascular risk [88], these anti-inflammatory effects of semaglutide may translate into additional cardiac benefits [66], [67], [87].

Furthermore, semaglutide modulates immune system activity by decreasing the recruitment or activity of immune cells [66], [68]. Rakipovski et al. demonstrated that semaglutide reduces leukocyte recruitment and rolling and decreases atherogenic plaque formation in mice [66]. Hansen et al. reported that semaglutide suppresses the recruitment of cytotoxic T-cells (CD8 + ) into hepatocytes in an animal model of non-alcoholic steatohepatitis (NASH) [54]. Other suggested mediating pathways by which semaglutide modifies immune system activity include reducing the proliferation of inflammatory cells [62], lowering the uptake of activated macrophages in blood vessels (resulting in fewer vascular injuries) [79], and reducing the development of atherosclerotic plaque lesions [66]. In summary, semaglutide can modify immune system function through various molecular mechanisms.

### Indirect pathways

Inflammatory processes can be activated in response to other pathways, such as oxidative stress [90]. Additionally, they are associated with pathological conditions such as obesity, which is characterized by underlying chronic inflammation [62]. There is evidence suggesting that semaglutide indirectly exerts anti-inflammatory effects by suppressing these mediating mechanisms [19], [62], [80]. It has been shown to reduce oxidative stress-dependent inflammation in H9c2 cells treated with LPS through an AMPK-dependent pathway, leading to decreased production of reactive oxygen species (ROS) and lower levels of NF-κB, TNF-α, and IL-1β [19]. Furthermore, semaglutide has been found to ameliorate obesity-induced inflammation by down-regulating S100a8, S100a9, and Cxcl2 in neutrophils of obese mice [62], [80]. It has also demonstrated a reduction in obesity-dependent inflammation in obese mice [91]. These effects may represent additional molecular links between semaglutide and the reduction of inflammation.

## Cardiovascular benefits of anti-inflammatory effects of semaglutide

**Fig. 2:**
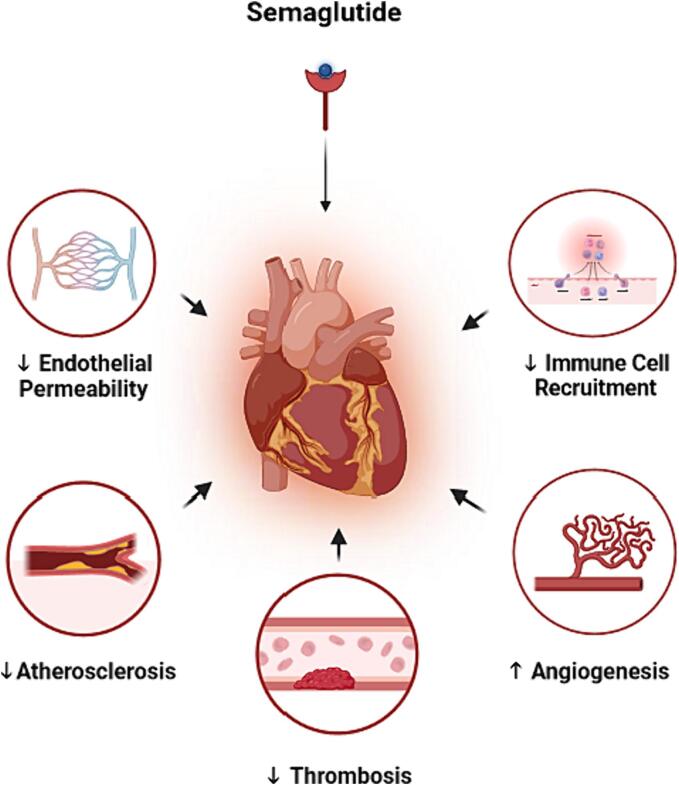
Semaglutide improves cardiovascular function by its anti-inflammatory benefits thru several mechanisms. It can preserves endothelial permeability, reduce immune cells recruitment into heart tissues, decrease atherosclerotic and thrombotic processes and induce angiogenesis in myocardium.

The anti-inflammatory effects of semaglutide have been shown to provide cardiovascular benefits in several experiments [62]. Semaglutide has been found to protect endothelial progenitor cells by inhibiting the expression of miR-155 (a microRNA) in macrophage exosomes [62]. MiR-155 induces inflammation in macrophage exosomes and impairs the function of endothelial progenitor cells, so its inhibition is associated with improved endothelial function [62]. Semaglutide has also been shown to improve the function of aortic endothelial cells and induce the angiogenesis process in the myocardium [67]. In various experiments, semaglutide has reduced myocardial injury and improved cardiac function by suppressing inflammatory responses [19], [62], [80]. These anti-inflammatory effects have resulted in pro-thrombotic, anti-atherosclerotic, and anti-atherogenic benefits in animal [66], [79], [86] and human [67], [80] studies. Furthermore, semaglutide may improve vascular structure and preserve endothelial permeability by normalizing the elements involved in the extracellular matrix and cytoskeleton, such as Coll5a1, Lama4, and Sparc [91], [92]. Therefore, it appears that semaglutide may protect the cardiovascular system, improve cardiac function, and promote ventricular thickening through its anti-inflammatory effects (Fig. 2) [79], [80], [93].

## Conclusion

Inflammation plays a significant role in the pathophysiology of diabetes and its associated complications, and controlling inflammation could be a major target for attenuating or preventing these disorders. Semaglutide, a long-acting GLP-1 analogue, has potent antidiabetic properties and normalizes glucose homeostasis through several pathways. Recent evidence suggests additional anti-inflammatory effects of semaglutide. While there is still limited available evidence, current knowledge suggests that semaglutide is able to reduce circulating inflammatory cytokines and modulate immune system responses. Further studies are needed to fully understand all the pathways involved, but current evidence strongly suggests cardiovascular and hepatic benefits for semaglutide based on its potent anti-inflammatory effects.

## CRediT authorship contribution statement

**Habib Yaribeygi:** Writing – original draft, Conceptualization. **Mina Maleki:** Writing – review & editing. **Tannaz Jamialahmadi:** Writing – review & editing. **Amirhossein Sahebkar:** Writing – review & editing, Conceptualization.

## Declaration of competing interest

The authors declare that they have no known competing financial interests or personal relationships that could have appeared to influence the work reported in this paper.
